# Supplementary material for: Benchmarking a fast, satisficing vehicle routing algorithm for public health emergency planning and response: “Good Enough for Jazz”
Source: PeerJ Comput Sci. 2023 Sep 1;9:e1541. doi: 10.7717/peerj-cs.1541 (PMC10495958; doi:10.7717/peerj-cs.1541)
Supplement: Supplemental Information 1 — Algorithm psuedocode for RSSD and a text description version of the RSSD flowchart in Figure 2. [file peerj-cs-09-1541-s001.pdf]

Appendix A. Supplementary Material on RSSD Algorithm

Algorithm 1 RSSD Algorithm

Input
V, D, p, τ, ψ
Output
R: $R_i \in R : \forall t(r_i) \leq \tau \wedge c(r_i) \leq \psi$

$tcr \leftarrow$ **tCompliant**(V, D, τ)
bool *noncompliance* $\leftarrow$ true
while *noncompliance* **do**
    $ctcR, pr \leftarrow$ **cCompliant**(tcr, D, ψ)
    $R \leftarrow R \cup ctcR$
    if $t(pr) > \tau$ **then**
        $mtcR \leftarrow$ **tCompliant**(pr, D, τ)
        $tcR \leftarrow tcr \cup mtcR$
    elif $c(pr) > \psi$ **then**
        $mtcR \leftarrow$ **tCompliant**(pr, D, τ)
    else
        $noncompliance \leftarrow false$
        $R \leftarrow R \cup pr$
    end if
end while

Algorithm 2 tCompliant

Input
V, D, p, τ
Output
tcR: $R_i \in R : \forall t(r_i) \leq \tau$

$r_1, r_2 \leftarrow$ **SpatialPartition**(V, D, p)
$tcR \leftarrow tcR \cup r_1, r_2$
while $t(r_i) > \tau \forall r_i \in tcR$ **do**
    $tcR \leftarrow tcR\ r_i$
    $r_a, r_b \leftarrow SpatialPartition(r_i, D, p)$
    $tcR \leftarrow tcR \cup \{r_a, r_b\}$
end while

Algorithm 3 SpatialPartition

Input

V, D, p

Output

tcR: $R_i \in R : \forall t(r_i) \leq \tau$

$r_a, r_b \leftarrow \{V \backslash p\}.maxVs(D)$
while $|r_a| + |r_b| \neq n - |p|$ **do**
  if $t(r_a) \leq t(r_b)$ **then**
    $r_a \leftarrow r_a \cup v_i$, where $v_i \leftarrow \{r_a^{-1}, r_a^0\}.minV(D, V \backslash \{p, r_a, r_b\})$
  else
    $r_b \leftarrow r_b \cup v_i$, where $v_i \leftarrow \{r_b^{-1}, r_b^0\}.minV(D, V \backslash \{p, r_b, r_a\})$
  end if
  $r_a \leftarrow r_a \cup p_x$ where $p_x \leftarrow \{r_a^{-1}, r_a^0\}.minV(D, p)$
  $r_b \leftarrow r_b \cup p_x$ where $p_x \leftarrow \{r_b^{-1}, r_b^0\}.minV(D, p)$
end while
return r_a, r_b

Algorithm 4 cCompliant

Input

$tcR, p, D\psi$

Output

$ctcR$: $R_i \in R : \forall t(r_i) \leq \tau \wedge c(r_i) \leq \psi$

pr: New route composed of pruned notes

while tcr **do**
  for r_i **in** tcR **do**
    while $c(r_i) > \psi \forall r_i \in tcR$ **do**
      $pr \leftarrow pr \cup v_i$, where $v_i \leftarrow p.minv(D, r_i)$
      $r_i \leftarrow r_i \backslash v_i$
    end while
    $ctcR \leftarrow ctcR \cup r_i$
    $tcR \leftarrow tcR \backslash r_i$
  end for
end while
return $ctcR, pr$

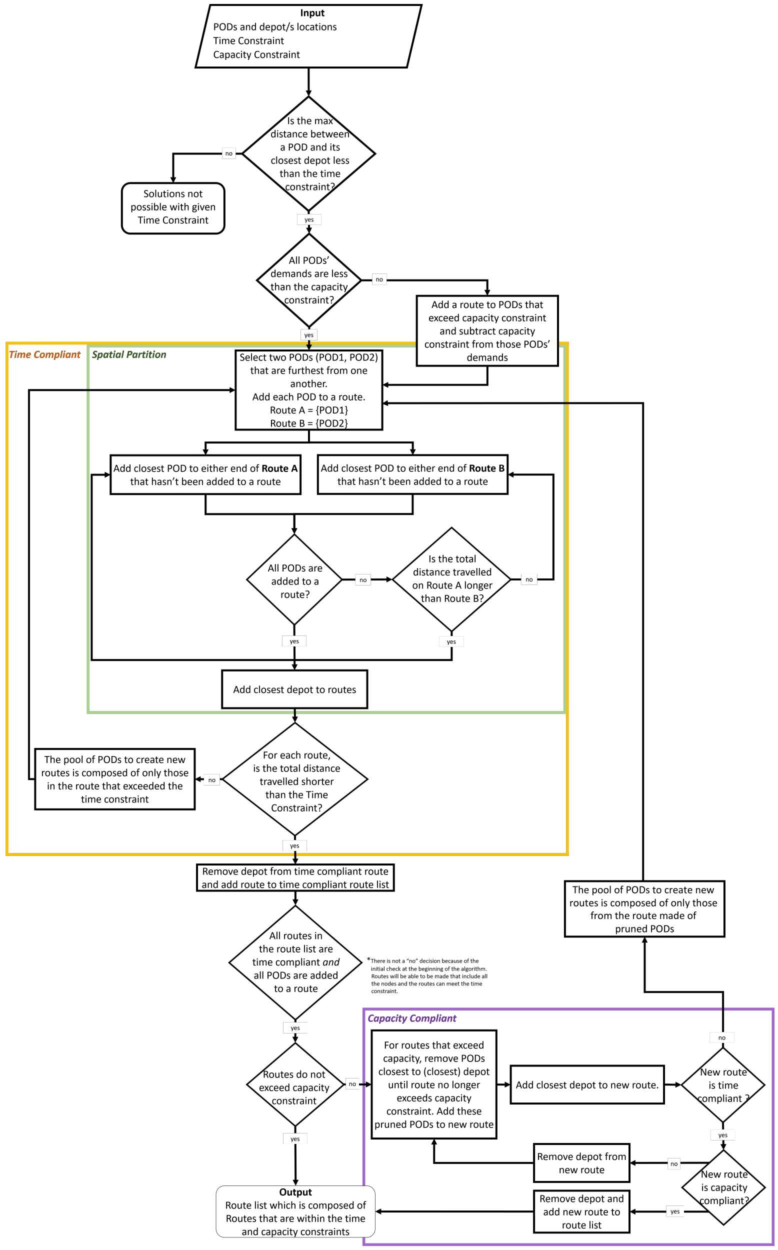

Figure A1 – The same flow chart as Figure 2 about the work flow of RSSD, but instead uses text explanations instead of mathematical notation.
