# Supplementary material for: Benchmarking a fast, satisficing vehicle routing algorithm for public health emergency planning and response: “Good Enough for Jazz”
Source: PeerJ Comput Sci. 2023 Sep 1;9:e1541. doi: 10.7717/peerj-cs.1541 (PMC10495958; doi:10.7717/peerj-cs.1541)
Supplement: Supplemental Information 2 — Two extra graphs of algorithm process times discussed in the Results. [file peerj-cs-09-1541-s002.pdf]

Appendix B. Supplementary Graphs of Process Times

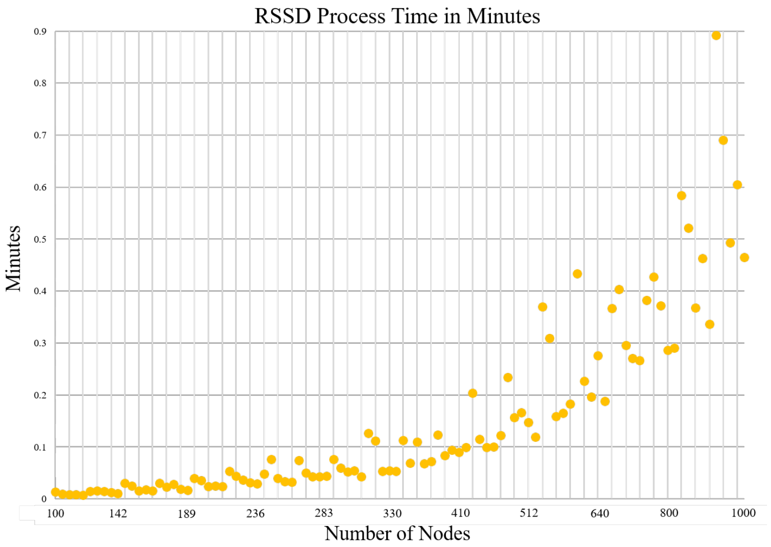

Figure B1 – The process time for RSSD across all instances in minutes

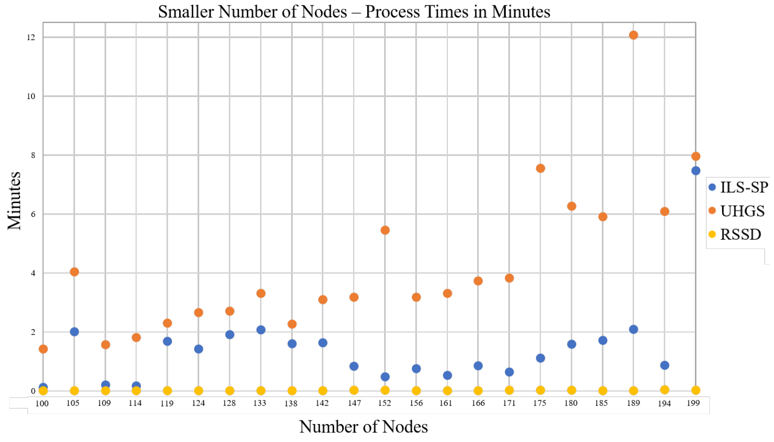

Figure B2 – The process time in minutes for ILS-SP, UHGS, and RSSD across instances with less than 200 nodes
