# Supplementary material for: Benchmarking a fast, satisficing vehicle routing algorithm for public health emergency planning and response: “Good Enough for Jazz”
Source: PeerJ Comput Sci. 2023 Sep 1;9:e1541. doi: 10.7717/peerj-cs.1541 (PMC10495958; doi:10.7717/peerj-cs.1541)
Supplement: Supplemental Information 3 — The distributions across all metrics (UnCap, RngMax, NoR) for the best known, RSSD, and the difference between the two. [file peerj-cs-09-1541-s003.pdf]

Appendix C. Data Table

Table C1 – Mean, Min, Max, and Standard Deviation across metrics for all solutions.

	Best Known				RSSD				Difference			
	Mean	Min	Max	SD	Mean	Min	Max	SD	Mean	Min	Max	SD
UnCap	0.0277	0.0000	0.1352	0.0268	0.2487	0.0398	0.8364	0.1414	-0.2210	-0.8162	-0.0382	0.1305
RngMax	0.8223	0.4751	0.9974	0.1180	0.7181	0.3349	0.9955	0.1638	0.1042	-0.2140	0.4973	0.1318
NoR	0.1267	0.0409	0.3333	0.0777	0.1524	0.0468	0.3881	0.0919	-0.0257	-0.0786	-0.0047	0.0195
